# Tests for the replication of an association between *Egfr *and natural variation in *Drosophila melanogaster *wing morphology

**DOI:** 10.1186/1471-2156-6-44

**Published:** 2005-08-15

**Authors:** Arnar Palsson, James Dodgson, Ian Dworkin, Greg Gibson

**Affiliations:** 1 Department of Genetics' North Carolina State University, Raleigh, NC 27695, USA; 2 Department of Ecology and Evolution, University of Chicago, Chicago, IL 60637, USA; 3 The Department of Biochemistry, University of Sussex, Brighton, BN1 9QG, UK

## Abstract

**Background:**

Quantitative differences between individuals stem from a combination of genetic and environmental factors, with the heritable variation being shaped by evolutionary forces. *Drosophila *wing shape has emerged as an attractive system for genetic dissection of multi-dimensional traits. We utilize several experimental genetic methods to validation of the contribution of several polymorphisms in the *Epidermal growth factor receptor *(*Egfr*) gene to wing shape and size, that were previously mapped in populations of *Drosophila melanogaster *from North Carolina (NC) and California (CA). This re-evaluation utilized different genetic testcrosses to generate heterozygous individuals with a variety of genetic backgrounds as well as sampling of new alleles from Kenyan stocks.

**Results:**

Only one variant, in the *Egfr *promoter, had replicable effects in all new experiments. However, expanded genotyping of the initial sample of inbred lines rendered the association non-significant in the CA population, while it persisted in the NC sample, suggesting population specific modification of the quantitative trait nucleotide QTN effect.

**Conclusion:**

Dissection of quantitative trait variation to the nucleotide level can identify sites with replicable effects as small as one percent of the segregating genetic variation. However, the testcross approach to validate QTNs is both labor intensive and time-consuming, and is probably less useful than resampling of large independent sets of outbred individuals.

## Background

Elucidation of the specific genetic variants that underlie natural phenotypic variation constitutes a major challenge for evolutionary geneticists. Our understanding of evolution will remain incomplete until the relative proportions of deleterious, (nearly) neutral and adaptive factors are documented, in terms of number of loci, their individual and joint effects as well as mode of expression [1]. Several practical issues complicate this endeavor. First, assessment of the contribution of loci and nucleotide variants can be confounded by chance effects, leading to inflated estimates [2]. Second, precise assessment of the effects of segregating polymorphisms on phenotypes depends critically on accurate mapping of the variants, down to individual quantitative trait nucleotides (QTN). Third, environmental interaction, epistasis and pleiotropy, all add complexity to the architecture of genetic variation[1,3].

Most common implementations of quantitative trait locus (QTL) mapping have low bias with respect to genomic coverage, but only identify allelic variation between two strains. In model organisms, these approaches allow assessment of marginal and epistatic effects, since the experiments are conducted with a large number of offspring, often in laboratory settings that reduce environmental variance. In practice, QTL are rarely resolved to individual loci or exact causal genetic variants [3-5], although several studies on plants offer exceptions [6,7] (reviewed in [8]). In *D. melanogaster*, QTL loci have also been dissected with quantitative complementation tests [9,10] and/or by linkage disequilibrium (LD) mapping involving a candidate region or locus. These approaches have the resolution to establish a significant contribution of allelic variation at single genes [9,11-20] and even specific nucleotides [21-23].

Successful implication of allelic and nucleotide variation in candidate genes in the production of phenotypic variation is aided by low amounts of LD, due to substantial historical recombination, in the fly genome. LD mapping in *D. melanogaster *can be implemented with varying degrees of control over genetic and environmental variance from wild caught individuals, laboratory reared iso-female lines, inbred strains, chromosome extraction lines and strains with introgressed chromosome regions. It is now clear that the power and resolution of association studies varies among organisms according to the extent of haplotype structure, and that different experimental approaches must be taken to verify associations in each organism. Despite the lesson from LD mapping in humans that extensive repetition, across cohorts and populations, is crucial to verify allelic contributions [24,25], replication of associations in model organisms is almost non-existent. More research into genetic approaches to validation of QTN effects is needed.

Drosophila wing shape has been used extensively as a model for the study of integration of developmental and quantitative genetics [26,27] and for analysis of the evolution of clinal variation in morphology [28-30]. More specifically, wing shape has proven to be an amenable system for studies on developmental modularity and integration [31], developmental stability [32], selection responses [33-35], laboratory adaptation [36] and more recently for the quantitative genetic dissection of patterning [23,37-41]. Wing shape is commonly described by geometric morphometric tools [42] that capture variation in the locations of landmarks at junctions of veins, cross-veins and the wing margin. The veins have a stereotypical configuration in the Sophophoran family of Drosophilids, with only minor differences documented between species [43], but diversity of shape is considerable [44,45]. Wing shape is highly polygenic [26,33,34,46] and we proposed that the spacing and length of veins is a major source of this variation [47].

QTL mapping and quantitative complementation tests support the involvement of venation loci, including components of the EGFR/Ras pathway, in naturally occurring wing shape variation [38,41]. These observations led us to test association between allelic variation in the *Egfr *locus and shape, by sequencing ~11 kb of the locus in 210 inbred lines from two North American localities, NC and CA [23,48]. Significant association of six polymorphisms in *Egfr *with aspects of wing shape and size, either as main effects or by interaction with population or sex, were reported. A follow-up with wild caught flies confirmed one of the associations, suggesting that QTN effects responsible for less than one percent of the variation for a complex trait can be isolated [49].

**Figure 1:**
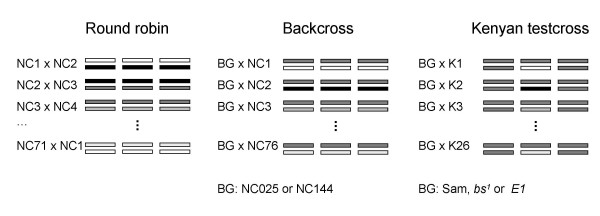
Schema of the three experimental crosses. (**A**) In the round robin (RR), each of 71 inbred lines from NC was crossed to six other lines to produce heterozygous offspring. Six loops of the type shown were used. (**B**) In the Backcross (BC), each of 76 NC lines were crossed to two phenotypically divergent backgrounds, NC025 and NC144, again resulting in heterozygous offspring. (**C**) Each of 26 Kenyan second chromosomes extracted into the Samarkand background were crossed to regular Samarkand or Samarkand lines carrying *blistered*^*1 *^or *Egfr*^*Ellipse *^mutations.

The aim of the current study was to assess the capacity of a series of controlled cross designs to validate the contribution of *Egfr *polymorphisms to naturally occurring variation for wing shape and size. Three schemes were employed, two involving crosses among a subset of the NC lines (a round robin in which 71 nearly isogenic lines were each tested in six random crosses to each other; and a backcross of each of 79 of the lines to two of the most phenotypically extreme lines), and a third involving test crosses between an independent set of Kenyan second chromosomes and the Samarkand wild-type and *Egfr*^*E1 *^and *blistered*^*1 *^mutant alleles (Figure 1). Only one of the six previously reported associations replicated in all datasets, the variant in the *Egfr *promoter that showed the most significant main effect in the original study and that also replicated in the wild caught flies [49]. However, when we increased the genotyping in the inbred lines, an interesting dichotomy appeared: the association persisted in the North Carolinian sample but vanished in the Californian population. These results argue for the need of large samples, direct contrast of genetic designs, and most importantly increased replication across populations to fully explore the utility of LD mapping to ascertain nucleotide differences affecting continuous variation of evolutionary importance. They also have implications for the fundamental question of whether quantitative genetic variants have variable effects in different populations [50,51].

## Results

### Similarity of shape variation between datasets

**Figure 2:**
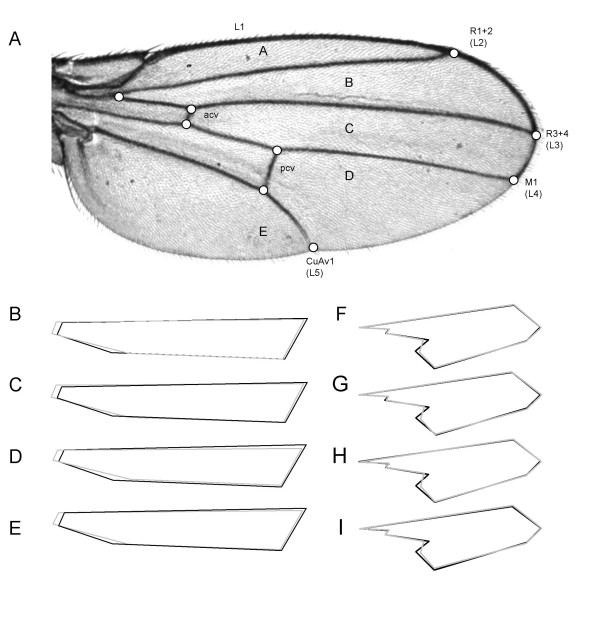
Shape of the *D. melanogaster *wing was captured from 9 landmarks (**A**) and analyzed with TPS software [68]. Veins are labeled both by the Comstock and Needham [69] nomenclature and using developmental genetic terminology (L1–L5). Individual inter-vein regions are designated by the letters A-E. (**B – I**): Shape differences derived from the four different datasets, namely the inbred (**B**, **F**), backcross (**C**, **G**), round robin (**D**, **H**), and Kenyan (**E**, **I**) panels. **B-E **shows the first principal component for landmarks of the central region of the wing (C1). The region is specified by longitudinal veins L3 and L4 and the placement of the cross veins and the margin. **F-I **show the shape differences for the seventh principal component for the whole wing data (W7). The whole wing and the central portion are not drawn to scale. Dark lines represent negative values, and gray positive. For whole wing PC 7 (W7) the extremes are at +/- 0.01 units and for central region PC 1 (C1) the values are +/- 0.2.

**Figure 3:**
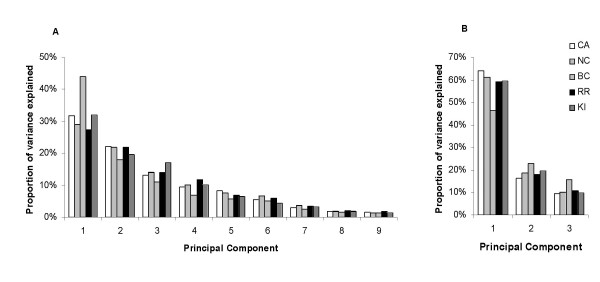
Eigenvalue distribution for the PC's, derived from each of the five datasets individually. (**A**) Values for the 9 PC's capturing variation in the whole wing, and (**B**) for the 3 PC's capturing variation for the five landmarks that define the central region of the wing. The decomposition of eigenvalues is comparable for all datasets, with the exception of the Backcross (BC) dataset which deviates qualitatively for the first component in both panels. To illustrate the consistency of shape capture, the principal components for the NC and CA populations were extracted individually for this Figure. All other analyses were conducted on PC's estimated for the CA and NC populations jointly.

Comparison of genotype-phenotype associations between datasets requires that the phenotypic measurements be comparable. We have adopted principal component (PC) descriptors of shape, and although these are modified subtly by inclusion of more wing data [23] overall the shape metrics extracted from each dataset individually are remarkably similar as depicted for consensus configurations of standardized principal component deviations in Figure 2B–I. This is true both for major (for example C1) and minor (W7) principal components, suggesting that shape variation in North American and African populations of *D. melanogaster *wings reduces to few shared dimensions (see also reference [52]). Furthermore, the eigenvalue decomposition for principal components derived from the individual experiments is qualitatively similar, as shown in Figure 3A. The only exception is the Backcross dataset, where the first PC's for the central region and the whole wing have unusually extreme values. This commonality of the axes of shape variation justified re-extraction of PC's for all datasets jointly, and these joint values were used for all subsequent tests of association. Note that the use of "jointly" or "separately" derived PC's has negligible effect on the test statistics for genetic terms and estimated effects (Table 1 and Additional table 1).

**Table 1:** Retesting the effects of *Egfr *SNP's on wing shape and size

				Separately derived ^a^				Jointly derived ^a^			
SNP	Term^b^	Trait	Type^c^	INB	RR	BC	KI	INB	RR	BC	KI
C31656T	Gtyp × Sex	Area	N	****	.	.	.	****	.	.	.
C30200T^d^	Gtyp	C1	N	****	**	****	***	****	**	****	***
T31634C	Gtyp × Sex × Pop	C2	N	****	.	.	.	****	.	.	.
T39389C	Gtyp	D1	S	****	.	.	.	****	.	.	.
T40722C	Gtyp × Pop	Area	S	****	.	.	na	****	.	.	na
C30505A	Gtyp	W7	N	***	***	*	**	***	***	**	*

a. As described in the Materials and Methods, PC's were calculated for the datasets individually (separately) or for all the data concatenated (jointly).

b. Term denotes the genetic term most significant in the original study. The same term is reported for the repeats, except for the RR where we could only test genotype effects and sites T31634C and T40722C where the genotype terms are reported as population terms are not available.

c. Type indicates the nature of the polymorphism, N: non-coding and S: synonymous.

d. The significance of the T30200C to C1 association is here reported for the data from Palsson and Gibson 2004. After re-genotyping the *p*-values reduce to 0.062 and 0.061 for the separately and jointly derived data respectively, when analyzed over the NC and CA populations. Note however that the results for RR and BC are for the re-genotyped data. Significance of terms: "." non-significant, "*": 0.05 > *p *> 0.01, "**":*p *> 0.001, "***":*p *> 0.0001, "****":*p *> 0.00001. *P*-values are not adjusted to correct for the seventeen new independent tests conducted.

### Absence of support for effects of *Egfr *on wing *size*

In order to re-evaluate our previously published associations between wing *size *and *Egfr *polymorphisms, recrossing of inbred lines used earlier and testcrosses of additional African chromosomes was carried out. Neither of the two variants affecting size of the wing (C31656T and T40722C) in the initial study gave a significant association in any of the three new datasets (Table 1: RR, round robin; BC, backcross; KI, Kenyan introgressions). In the initial study, polymorphism C31656T had the strongest association, a Genotype by Sex interaction (*p *= 0.000002) that also exhibited a possible three way interaction of Population, Sex and Genotype (*p *= 0.001). As the three-way interaction was primarily caused by larger difference in the CA than the NC sample [23], the lack of signal in the crossed NC lines is not surprising. Similarly, while T40722C had previously opposite effects on size depending on population, its contribution in the NC population was neither replicated in the BC and RR recrossing experiments nor in the Kenyan sample. These results indicate that the previously reported association of *Egfr *with wing size was likely a false positive even though it was significant after adjustment for the number of multiple comparisons experiment-wide.

### Replicable effects of one *Egfr *variant on wing *shape*

The two crossing schemes and the Kenyan introgressions were used to re-evaluate the contribution of four *Egfr *variants to aspects of wing *shape*. Only one polymorphism T30200C, was significant and had consistent effects in all of these experiments. This variant resides in the second alternate promoter in a putative GAGA factor binding site, and contributes to the first principal component of the central region of the wing (C1: Table 1 and Figure 2B–E). One other polymorphism, C30505A in the same promoter, was also significant in all experiments, but had opposite effects on shape metric W7 in the Kenyan sample compared to the Inbred, BC and RR experiments. The inconsistency of the effects casts serious doubt on this association.

Neither of two other previously reported putative associations [23], the sex and population dependent contribution of site T31634C to the width of the central region (C2) nor the contribution of T39389C to the posterior region (D1) were supported by the new data. The lack of association of T39389C prompted us to re-examine epistatic effects which included this particular site and associated with variance in the posterior region (D1) of the wing [23]. The three site *Egfr *haplotype (G6065T, T39389C, and T40110C) and also each of the two site haplotypes had given highly significant association in the original panel of inbred lines. Due to smaller sample size in our recrossing datasets, testing of this pattern could only be conducted with the BC dataset, but the previous epistatic interactions were not confirmed (data not shown). In summary, only one of the *Egfr *polymorphisms previously implicated to impact wing shape was corroborated by the new data.

### Breakdown of the T30200C association in the Californian population

**Figure 4:**
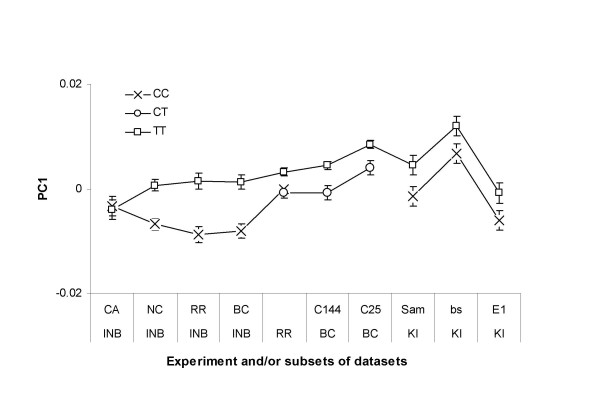
Effects of the T30200C polymorphism on C1 in females across experiments and genetic configurations (designated on the X-axis). INB refers to the inbred populations CA and NC, while INB_RR and INB_BC denote the subsets of inbred lines corresponding to the lines used for the recrossing experiments. Likewise, C144 and C25 indicate the estimated effects of the site in backcrosses to line NC144 and NC025 respectively. The last three points show the effects estimates for the three test crosses involving the Kenyan introgressions (KI), namely wildtype Samarkand chromosomes, the *blistered*^*1 *^mutant, and the *Egfr*^*Ellipse *^allele. Each point represents the least square estimate plus or minus one standard error unit for the indicated homozygous genotypes. See Additional Table 2 for corresponding ANOVA's and values for tests with the older genotype data. The PC's were extracted from all datasets jointly to ensure that the axis of variation and units are comparable.

Previously, due to incomplete genotyping around exon 2, the contribution of T30200C to the central region of the wing was only evaluated with 79 NC and 43 CA lines [23]. Analyses by population found highly significant association in the North Carolinian sample (*p *= 0.00002) but only marginal association in the west coast sample (*p *= 0.04) (see Additional Table 1). In order to obtain a better estimate of the magnitude of the effect of T30200C on cross-vein placement, and to investigate the apparent difference in effect between populations, extra genotyping was conducted. The sampling of this polymorphism was increased by re-genotyping the surviving lines from the two populations. Repeating the analysis of variance with 121 NC lines reduced the significance of the association of the T30200C polymorphism (*p *= 0.002). More dramatically, the addition of 30 more alleles to the CA lines (N = 76) rendered the originally marginal association non-significant (*p *= 0.9) (Additional Table 1). Inspection of estimated genotypic effects demonstrates this clearly (Figure 4 and Additional Table 2), as the homozygous classes have nearly identical values for the CA population. Evaluation of the effect of this site in the full dataset without population as a term in the model also renders the association non-significant (*p *> 0.05).

### Magnitude of the *Egfr *allelic contribution

Estimates of the genotypic effects of T30200C on wing shape are comparable across all of the datasets. There was a slight reduction in observed contribution after the extra genotyping (Additional Table 2), and the estimated difference between homozygote classes was smaller in the RR data than in the NC lines, with the CC and TC heterozygotes being indistinguishable. This suggestion of dominance is opposite that observed in a large sample of outbred flies [49] in which heterozygotes resembled TT homozygotes (dominance was non-zero in this study), but it should be noted that CC homozygotes are very infrequent in the current study. In the BC experiments, only TT and TC genotypes were available but the magnitude of the difference between genotypic classes was nearly identical in both backcrosses (to NC025 and NC144) and in the RR experiment (Figure 4 and Additional Table 2). The general differences were again of the same magnitude and direction in the testcrosses involving the Kenyan chromosomes, and they scaled additively with the genetic background (Samarkand, E1 or *bs*^*1 *^carrying chromosomes).

### Experimental designs and potential sites with weak effects

**Figure 5:**
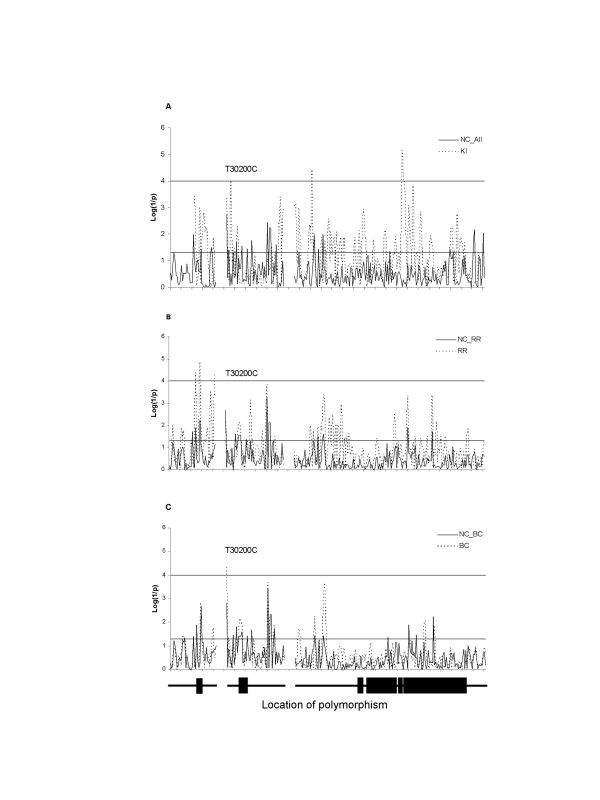
Association plots for tests of association between *Egfr *and shape parameter C1. Each plot shows the negative logarithm of the *p*-value for the test statistic at each polymorphic SNP from 5' to 3 along the *Egfr *locus. (**A**) Association profile from the whole NC inbred panel (N = 121, solid line) and the Kenyan chromosomes (N = 26, broken line). (**B**) The association profile for the RR experiment (outcrossed, N = 71, broken line) and the subset of NC lines (in inbred condition, N = 71, solid line) used for the outcrossing scheme. (**C**) Similarly the profiles for the BC panel (backcrossed to NC025 and NC144, N = 79, broken line) and the corresponding NC set (N = 79, solid line). The X scale is broken to indicate gaps between contigs with the gene structure represented below (exons as boxes in three contigs of non-coding sequence). Site T30200C (indicated) is located at the 5' most end of contig 2. Lines corresponding to single site significance levels α = 0.05 (negative log *p*: 1.30) and more conservative gene wide α = 0.0001 (negative log *p*: 4.0). Trait values for all lines and crosses come from data that were processed jointly in TPSrelw [68].

In order to compare the gene-wide patterns of association for each design, the association statistic for the Genotype effect of each site along the *Egfr *locus is plotted for the three experiments in Figure 5. In each plot, higher significance is toward the top, with thresholds drawn at *p *= 0.05 and *p *= 0.0001 as before [23]. The analysis focuses on the effects on trait C1, on basis of the assumption that the T30200C association implicates this shape metric as being affected by variation in *Egfr*.

The first general result is that the small sample of Kenyan introgressions provides more highly significant sites than the total NC sample (with the exception of T30200C there are no significant associations in common between these two populations). Similarly the RR design yielded more significant test statistics (three sites in the first exon) then the BC or inbred panels and had 55 sites exceeding the test-wise significance threshold of *p *= 0.05. The observed jaggedness of the association profiles likely reflects stochastic fluctuations in the *p-values *in experiments with relatively small sample size. One interpretation of the data is that the inbred and backcross designs provide better dampening of this stochastic fluctuation then do studies with round robin crossed inbred lines.

The second result is that, in both the RR and BC experiments, the shape of the association profile tracks quite closely with that of the corresponding profile for the set of nearly isogenic lines used to set up the testcrosses. This was not anticipated, since NC025 and NC144 lines have very different wing shapes and each contribute 25% of the genetic variation in the BC, while the RR combines the genetic variation of the 71 inbred lines in equal proportions. Evidently genetic correlations between the different testcrosses are sufficient to produce similar association profiles, whether or not these accurately report QTN effects.

Finally, in order to test whether other sites in *Egfr *affect the cross-vein placement we performed a combined Mixed model ANOVA on the three NC datasets (NC, RR and BC). Eleven independent polymorphisms summarized in Table 2 were observed to be significant at the experiment-wide significance level of *p *< 0.0001, including site T30200C. Most of these sites are not significant in the CA and Kenyan datasets, but the direction of the genotypic effects generally correspond with the NC panels (only 2/14 are non-concordant, one tailed Fisher exact test yields *p *= 0.052). Only one of these new candidate variants, C6085G in the less conserved of the two alternate first N-terminal exon, alters the protein, while the remaining are non-coding or silent. Interestingly, one of these silent polymorphisms is C40620T, which also associates with cryptic variation for eye-roughness in inbred lines and wild flies [21]. Note however, if the *Egfr *variants are tested against other principal component measures of wing shape, similar number of sites emerge at the level of *p *< 0.0001 (data not shown) suggesting the caveat that this approach may be inherently noisy.

**Table 2:** Significance of *Egfr *polymorphisms on central region shape in NC, CA and Kenyan samples

				Effect and significance^c^		Frequency of rare allele		
				
Site	Significance in NC	Location^a^	Type^b^	CA	KI	NC	CA	K
C6085G	7.98 × 10^7^	E1	R	(OK) ^ns^	(Rev) ^ns^	15/125	13/75	4/27
T30200C	6.11 × 10^10^	P2	N	(Rev) ^ns^	(OK) ***	26/121	21/76	8/17
A31442T	5.49 × 10^8^	I2	N	(OK) ^ns^	(OK) ^ns^	13/92	3/24	2/20
A36644T	9.60 × 10^7^	I2	N	(Rev) ^ns^	(OK) *	26/116	3/20	9/24
A36761C	5.17 × 10^6^	I2	N	na	na	35/84	na	na
Del37192^d^	3.44 × 10^5^	I2	N	(OK) ^ns^	(OK) ^ns^	8/106	10/77	7/24
A37282G	9.95 × 10^6^	I2	N	nd	nd	13/106	1/77	0/23
T39160C	2.10 × 10^5^	E4	S	(OK) ^ns^	(OK) #	41/128	26/76	16/35
In39534^d^	8.81 × 10^5^	I5	N	(OK) ^ns^	(OK) ^ns^	27/110	6/27	3/33
C40620T^e^	7.37 × 10^7^	E6	S	(OK) ^ns^	(OK) #	46/123	19/79	7/36
G42242A	8.88 × 10^5^	3' UTR	N	nd	nd	5/105	1/12	1/25

a. Location within the *Egfr *locus, where P2 refers to the second promoter and the other indicators to the respective introns (I), exons (E) and the 3'UTR.

b. Type indicates the nature of the polymorphism; R: replacement, N: non-coding and S: synonymous.

c. Effects of the polymorphisms in the same direction as in NC are indicated by "OK" and those in the reverse direction are designated by "Rev", with the significance of the genotypic term indicated. "ns": not significant, "#:" 0.1 > *p *> 0.05. "*": *p *> 0.01, "**": *p *> 0.001, "***": *p *> 0.0001. *P*-values are not adjusted to correct for the number of tests conducted. "na": genotypes not available, "nd": not computed because of allele rarity.

d. Del37192 is a one base pair deletion and In39534 a four base insert (AACC repeated).

e. Site C40620T is the same as site 8697 described by Dworkin et al. [21].

## Discussion

Previously, fine mapping of the association between polymorphisms in the candidate locus *Egfr *and wing shape and size in *D. melanogaster *in 210 inbred lines from two North American populations [23] implicated six *Egfr *variants or linked polymorphisms as causal variants. In this study we aimed to re-evaluate their involvement through further genetic analysis by generating heterozygous lines derived from crosses of a subset of the original lines and by test crosses with a small sample of African chromosomes. Only one of the retested variants was significant in all datasets and gave consistent effects: the T30200C polymorphism that affects a principal component capturing variation in relative distance between the two cross-veins. However, even the estimated absolute magnitude of this effect is dependent on the survey population and crossing scheme. These results highlight the difficulties in validating weak quantitative effects through experimental genetic approaches and suggest that resampling of outbred populations may be the more conclusive approach to dissection of QTL to the nucleotide level.

### The T30200C association persists

There are at least three possible explanations for the observed restriction of statistical support for the association of T30200C with wing shape to just two of the three populations sampled. The first is that the observed associations in NC and Kenyan samples are false positives, namely that T30200C or linked variants in *Egfr *do not contribute to shape of the central region of the wing. This seems unlikely, since significant association was also observed in a large sample of outbred NC flies [49] and the association was also replicated in both of the testcross experiments described here.

Two alternative explanations are consistent with the statistical significance being indicative of a true contribution of *Egfr *polymorphisms to wing shape in NC. One is that the effect of T30200C is masked by genetic variation that is unique to the CA population. Another possibility is that T30200C is not the real causative variant, but exhibits high LD with the causative site in the NC and Kenyan populations but weak LD in the CA population. Since LD in the *Egfr *decays to background levels over several hundred bases and no differences were observed between NC and CA in their patterns of LD or allele frequencies, while both North American populations diverge considerably from the Kenyan sample [48], this latter explanation is also unlikely. T30200C does not differ in frequency between NC and CA (Fisher's exact test, *p *= 0.88), but it does lie adjacent to a 23 kb intron that has not been sequenced in the population sample and could conceivably harbor the true causative variant. However, we favor the hypothesis that one or more modifier loci that differentiate the two North American populations mask the expression of variation due to the *Egfr *in the CA sample.

Two developmental genetic arguments also lend support to the hypothesis that the T30200C variant is the causal site. First, our prediction that this site affects a GAGA factor binding element in the *Egfr *promoter, is supported by genetic interaction between the two loci [53,54]. Second, the association between *Egfr *and cross vein placement is in accord with developmental genetic evidence. Specifically, flies heterozygous for different *Egfr *alleles lack the majority of the L4 vein and the entire proximal cross-vein [53,55,56]. Recall that shape changes corresponding to principle component C1 for the central region of the wing (Figure 2B–E) represent variation in the distance between the cross-veins, both of which connect with vein L4.

### Detection of natural alleles with subtle effects

Quantitative traits in *D. melanogaster *are now being dissected with QTL mapping, quantitative complementation tests and by testing specific allelic variants by LD mapping. While several studies have found significant association between markers in candidate gene regions and continuous phenotypes [9,11-20] direct re-evaluations of these relationships remain rare. Mackay and Langley [18] found that large insertions around the *achaete-scute *locus influence bristle number, and this inference was corroborated in a second sample [16]. Geiger-Thornsberry and Mackay [57] confirmed the involvement of two previously identified *Delta *polymorphisms [15] on bristle number when the same flies were reared under different environmental conditions. Also, we found that three tightly linked silent *Egfr *polymorphisms affect cryptic variation in eye roughness in inbred lines, and then confirmed the finding in an independent sample of wild caught flies [21]. These studies corroborate the involvement of allelic variation in specific genes with quantitative traits. On the other hand, MacDonald and Long [58] failed to confirm the involvement of a large indel in the 5' region of *hairy *on bristle number that was previously observed [20]. Moreover, even though both Lai et al. [12] and Lyman et al. [17] implicated *scabrous *in variation for bristle number, these two studies differed in which markers were typed and by criteria for evaluation of significance (Lai et al. [12] reported an excess of associations with *p*-value below 0.05 while Lyman et al. [17] found three individual significant sites after permutation testing). Finally Genissel et al. [59] asked if the reported *Delta *bristle association [16] was caused by common replacement polymorphisms in the gene but were not able to identify the hypothesized causal variant.

In summary, several studies have aimed to validate the contribution of allelic to phenotypic variation, but interpretation is complicated by numerous differences between the studies, including: which population is sampled, the genetic designs, the types of genetic markers employed, and control over environmental variation. Additionally, while negative or only weakly suggestive results are sometimes reported [58-61], bias towards publication of positive results may prevent honest evaluation of the nature of the genetic basis of quantitative traits. In theory, once particular polymorphisms have been associated with an evolutionarily important trait, experimental genetic approaches can be used to confirm the functional differences between alleles [62-66]. However, due to technical complexity such methods have yet to be deployed to systematically gauge the effect of segregating variation in *Drosophila*. In the case of the *Egfr*, the proposed regulatory regions are too extensive to evaluate the dynamic contribution of allelic variants to vein and intervein determination, so extensive replication is the only viable approach to dissection of QTN effects.

### Mapping resolution and experimental designs

Successful fine mapping of QTL depends on multiple factors such as the magnitude of effect, pattern of LD in the region, available genetic resources, appropriateness of the selection of candidate genes/regions/molecular markers, and the dependence of expression of genetic variation on the experimental settings. The experiments reported here were designed to evaluate the potential for defined crosses to further dissect the role of QTN in subtle quantitative variation, but no obvious recommendations (apart from the need for deep sampling) are forthcoming since the different approaches only produce broadly comparable results.

The round robin and backcross approaches were designed to evaluate the degree to which effects observed in inbred lines are also seen in mixed genetic backgrounds. If the effects of the SNP are additive and there is no epistasis, then they should be just as strong in the testcrosses as in the nearly isogenic lines, with the caveat that there are three genotypes at each SNP to compare instead of just two. The BC design differs in two distinct ways from the RR design, namely the reduced genetic variation (two genomes contribute 50% of the alleles) and the capacity to detect epistatic effects. This latter could occur by interaction between the QTN and other loci, either due to de-canalization as these other loci perturb the phenotype away from the population mean, or simply because QTN effects may generally be so modified by the background that they are only observed in certain backgrounds. The similarity of the estimated genotypic mean differences over the two BC backgrounds and the close tracking of means in the KI experiment (Figure 4), suggests that the reduced genotypic variance is responsible for higher significance of the T30200C association in the BC cross. While this argues for the additivity of the genotypic effects in this case, it is not clear that similar effects will be observed for other traits or loci.

While the ten new highly significant sites in the combined model may be false negatives in the initial lines, more data would be required to confirm that they are true positives. These results indicate that recrossing and deeper population sampling has at best low power to detect novel candidate sites with subtle effects on the phenotype. Consequently, the testcrosses do not obviously outperform the inbred line analysis or bring us any closer to resolving true positive QTN from false positives. Even with a relatively large experiment such as this, the amount of labor and time spent on setting up several hundred crosses and phenotyping several thousand wings does not overcome sampling biases. Even if our analyses suggest that other sites in *Egfr *may affect cross-vein placement, a considerably larger sample than explored here would be required to validate these sites. The testcross results strongly suggest that we can eliminate highly significant results from the first experiment as false positives, but can not conclusively resolve the question of whether the *Egfr *QTL resolves to a single or several QTN.

## Conclusion

The *Egfr *contribution to shape variation in *D. melanogaster *wings reported in Palsson and Gibson [23], and replicated here and in Dworkin et al. [49], represent the best validated example of allelic contribution to continuous morphological variation in flies. While we can not assert that the polymorphism implicated is the causative variant, the evidence and literature cited provide hypotheses testable with experimental genetics. The practical lesson from the observation that five of the six retested *Egfr *variants failed to validate in testcrosses is that stochastic factors have a substantial impact on analysis of the genetic basis of continuous phenotypes in studies involving fewer than 200 inbred lines. Apparent conditional polymorphisms may be especially sensitive to these effects of chance, and all unreplicated association studies in *Drosophila *should be considered with this caveat in mind. We suggest that measurement of a very large number of offspring is essential for replication and validation in association studies, and that these are better sampled in outbred wild individuals than in laboratory lines. The declining cost of genotyping will facilitate this transition to large scale mapping of quantitative traits to single nucleotides in ecological settings.

## Methods

### Stocks and crossing schemes

Three separate experiments were conducted to re-evaluate the contribution of *Egfr *on wing shape (Figure 1). Two involved recrossing, by round-robin (RR) and modified backcross (BC) designs, (71 and 76 NC lines respectively, with 70 being shared). The RR crossing scheme is a partial diallele cross, with the 73 lines being crossed three times as sire and three times as dam. The mating scheme was derived by permutation. In the BC design, males from 76 NC lines were crossed independently with females from two strains NC025 and NC144. These inbred lines have extreme PC1 values for the anterior and posterior regions of the wing. The third experiment (KI) involved an independent set of Kenyan alleles from Ron Woodruff [48]. Second chromosomes were substituted into the Samarkand background by a 4 generation crossing scheme, utilizing stocks kindly provided by Trudy MacKay. Similarly an *Egfr *allele, *Ellipse *(E1), and a *blistered *allele (*bs*^*1*^) were substituted into Sam. The wild-type chromosomes were tested over these two mutations and the wild-type Samarkand second chromosomes in three replicate crosses arranged in random blocks. All crosses involved three males crossed to three females, and where conducted in two (RR and BC) or three replicates (KI).

### Fly rearing and scoring of wings

Flies were reared at 25°C in standard cornmeal medium with a constant light/dark cycle. Density was controlled by placing two virgin females and two males in a vial and discarding parents on the 2^nd ^or 4^th ^day depending on visual assessment of egg density. The right wing of eight to ten randomly selected individuals per sex (except only RR females) from each vial was scored. In the Kenyan introgression experiment the visible marker *Cy *on the balancer chromosome distinguished genotypes of lines inviable as 2^nd ^chromosome homozygotes. Handling of specimens and data processing was identical to previous experiments [47]. In short, wings were dissected at the hinge and arranged on glass slides and held in place with a cover slip. Within 48 hours, wings were digitally photographed at 4× magnification with a Spot camera, mounted on a Nikon microscope. Images were processed with Adobe Photoshop version 5, and landmarks captured in Scion Image (freeware available [67]). The nine landmarks at the junctions of veins and wing margin are depicted in Figure 2A. One author, JD, digitized the back-cross and ~65% of the round-robin while the remaining specimens (35% of RR, Inbred and Kenyan) were scored by AP. No significant "investigator" effects were found in an analysis of 1000 RR wings scored by both authors (not shown).

### Extracting common axes of shape variation

Shape variation was summarized with the TPSrelw software version 1.39 (freeware available [68]) by calculating relative warps for a set of landmarks, for the whole wing or individual regions (Figure 2A). The procedure involves "partial Procrustes" superimposition, by iterated rotation and alignment of specimens, rescaling to unit size, prior to extraction of the relative warps. The relative warps are essentially principal components (PC's), and will be referred to as such henceforth.

### *Egfr *genotype matrix

Genotypes used for the association tests were derived from our earlier sequence data [48]. The BC and RR recrossing was not designed to test particular polymorphisms, and therefore generated heterozygotes and sometimes both homozygotes at particular nucleotide positions. For instance in the BC design, of the six sites retested, T31634C and C30505A were not typed in NC144. Furthermore, of the remaining four polymorphisms, the lines differed only at T40722C. Note this does not mean that their *Egfr *haplotypes are highly similar, as 167 out the 232 common *Egfr *sites genotyped in both lines differ, with several recombination events evident. F1 lines that were missing a genotype of one parent where omitted from the analysis for that particular genotype. In the Kenyan sample, only the variant T40722C was not tested, as it was only available in one Kenyan line. The *Egfr *alleles were not sequenced in the three tester chromosomes, leading to tests on haploid data.

### Re-genotyping of T30200C

The T30200C polymorphism in the non-coding region upstream of alternative exon one [48] was re-genotyped in the NC and CA lines in 2004. The previous sample was incomplete due to high level of PCR failure that we attributed to repetitive elements in the region [48]. Therefore an alternative strategy for genotyping was deployed, utilizing the observation that this polymorphism affects a Restriction Length Fragment Polymorphism (RFLP) for the DraIII restriction endonuclease. As before, a single male from each line was genotyped [48]. For PCR, the following new primers were utilized as described in [49]: 5'-GTGGCTCGTAATGTGAAACT-3' and 5'-GCGTTACTGGTGGGATGAATCAAG-3'. Of the 210 original lines characterized in 2001–2002, 198 were still surviving in 2004 and were regenotyped. Three discrepancies were found, all in the NC panel (NC065, NC075, NC116). In the case of NC065 heterozygosity for the 3'end of the locus was noted in the original study and it is consequently quite possible that two alleles were segregating when the line was initially genotyped. Contamination of either DNA samples or stocks maintained over this period are also formal possibilities, particularly for the other two lines. These three lines were dropped from the re-analyses.

### Analysis of phenotypic variation

All statistical analysis used SAS version 8.2 (SAS Institute, Cary, NC). The estimation of line effects and extraction of line means was implemented with the LSMEANS option in Proc GLM. The model for the RR dataset was:

Y = *μ *+ Line + Rep(Line) + ε

where Line represents each of the F1 lines generated by the round-robin crosses, and Rep the replicate vial. For the Back-cross and the Kenyan introgression, a more complicated model was used, accounting for the effects of Cross (to NC025 and NC144 or to Sam, E1 and *bs*), Sex or Line.

Y = *μ *+ Cross + Sex + C × S + Line + S × L + C × L + C × S × L + Rep(C × L) + S × R(C × L) + ε

In both models terms including Line and Rep are considered random. We also performed the analysis without Rep as a term, with the same results.

### Tests of quantitative nucleotide effects

The main aim of these experiments was to re-evaluate the six sites which gave significant signals for wing size and shape in [23]. The RR experiment focused on females from a single population (NC) and a simple model was implemented in Proc Mixed:

Y = *μ *+ Gtyp + Rep(Gtyp) + ε

Gtyp is the fixed effect of Genotype, and Rep is a random term, again the replicate vials. For the back-cross and the Kenyan test cross, the model accounted for the contribution of sex and cross:

Y = *μ *+ Gtyp + Sex + Cross + G × S + G × C + G × S × C + Line(G × C) + ε

The mean effects of polymorphisms were estimated by the LSMEANS option. Reduced models, by crosses, and extended, by including replicates were also studied and were in accord.

In order to gauge the effects of additional sites in *Egfr *on the C1 we utilized a related model, substituting the Cross term with a fixed experiment (Exp) term to demarcate the NC, BC and RR datasets, and restricting the analysis to females as the RR panel had no males. The sire and dam are random effects nested within the fixed effects:

Y = *μ *+ Gtyp + Exp + G × E + dam × sire(G × C) + Rep(dam × sire × G × C) + ε

Sites with probability of genotype term below 0.0001, where then investigated for consistency in genotypic effects and their significance in the CA and KI dataset.

## Authors' contributions

AP, GG and JD designed experiments. JD crossed and scored RR and BC experiments, AP crossed and scored KI, Inbreds and parts of RR dataset. AP conducted statistical analysis. ID regenotyped the T30200C variant. AP, ID and GG wrote the manuscript and all authors approved the final version.

## Supplementary Material

Additional table 1 ANOVA tables for site T30200C and wing shape. The file shows the results of Analysis of Variance for the T30200C variant in the *Egfr *promoter and the first principle component for the shape of the central region of the wing.
Click here for file

Additional Table 2 Genotypic effects of T30200C on the first PC of the central region of the wing. This table illustrates the genotypic effects (and standard errors) for the T30200C association to wing shape of the central region.
Click here for file
