# Supplementary material for: Tests for the replication of an association between Egfr and natural variation in Drosophila melanogaster wing morphology
Source: BMC Genet. 2005 Aug 15;6:44. doi: 10.1186/1471-2156-6-44 (PMC1208880; doi:10.1186/1471-2156-6-44)
Supplement: Additional table 1 — ANOVA tables for site T30200C and wing shape. The file shows the results of Analysis of Variance for the T30200C variant in the Egfr promoter and the first principle component for the shape of the central region of the wing. [file 1471-2156-6-44-S1.doc]

## Additional Table 1. ANOVA tables for site T30200C and wing shape.

|  |  | Jointly derived PC’s^a^ | | | | Separately derived PC’s^a^ | | | |
| --- | --- | --- | --- | --- | --- | --- | --- | --- | --- |
|  |  | Partial sample ^b^ | | Extended sample ^b^ | | Partial sample ^b^ | | Extended sample ^b^ | |
| Exp. | Term | F(df,df)/  Est(SE)^c^ | *p-value* | F(df,df)/  Est(SE) ^c^ | *p-value* | F(df,df)/  Est(SE) ^c^ | *p-value* | F(df,df)/  Est(SE) ^c^ | *p-value* |
| INB_CA | Gtyp | 4.46_(1,42)_ | 0.040671 | 0_(1,72)_ | 0.980606 | 4.38_(1,42)_ | 0.042372 | 0_(1,71)_ | 0.973266 |
|  | Sex | 14.15_(1,42)_ | 0.000516 | 39.33_(1,72)_ | <0.000001 | 10.78_(1,42)_ | 0.002074 | 31.92_(1,71)_ | <0.000001 |
|  | G X S | 0.34_(1,42)_ | 0.564589 | 0.92_(1,72)_ | 0.340002 | 0.37_(1,42)_ | 0.548677 | 1_(1,71)_ | 0.321791 |
|  | *VC_line_* | 0.152(0.03) | 0.000004 | 0.179(0.03) | <0.000001 | 0.152(0.03) | 0.000004 | 0.179 (0.03) | <0.000001 |
|  | *VC_error_* | 0.006(0) | 0.000002 | 0.006(0) | <0.000001 | 0.006(0) | 0.000002 | 0.007 (0) | <0.000001 |
| INB_NC | Gtyp | 20.32_(1,77)_ | 0.000023 | 10.19_(1,114)_ | 0.001829 | 20.29_(1,77)_ | 0.000023 | 10.2_(1,114)_ | 0.001812 |
|  | Sex | 10.27_(1,77)_ | 0.001971 | 20.46_(1,114)_ | 0.000015 | 6.98_(1,77)_ | 0.009969 | 15.4_(1,114)_ | 0.000149 |
|  | G X S | 0.01_(1,77)_ | 0.932282 | 0.58_(1,114)_ | 0.44938 | 0_(1,77)_ | 0.948526 | 0.53_(1,114)_ | 0.466705 |
|  | *VC_line_* | 0.099(0.02) | <0.000001 | 0.112(0.02) | <0.000001 | 0.099(0.02) | <0.000001 | 0.113 (0.02) | <0.000001 |
|  | *VC_error_* | 0.007(0) | <0.000001 | 0.007(0) | <0.000001 | 0.007(0) | <0.000001 | 0.007 (0) | <0.000001 |
| INB_RR | Gtyp | 26.09_(1,52)_ | 0.000005 | 10.26_(1,68)_ | 0.002067 | 26.09_(1,52)_ | 0.000005 | 10.38_(1,68)_ | 0.001955 |
|  | Sex | 1.14_(1,52)_ | 0.289737 | 7.2_(1,68)_ | 0.009127 | 0.39_(1,52)_ | 0.534916 | 4.96_(1,68)_ | 0.02929 |
|  | G X S | 0.33_(1,52)_ | 0.567041 | 0.27_(1,68)_ | 0.605173 | 0.36_(1,52)_ | 0.551346 | 0.24_(1,68)_ | 0.623084 |
|  | *VC_line_* | 0.08(0.02) | 0.000001 | 0.116(0.02) | <0.000001 | 0.08(0.02) | 0.000001 | 0.116 (0.02) | <0.000001 |
|  | *VC_error_* | 0.007(0) | <0.000001 | 0.008(0) | <0.000001 | 0.007(0) | <0.000001 | 0.008 (0) | <0.000001 |
| INB_BC | Gtyp | 29.34_(1,57)_ | 0.000001 | 10.87_(1,74)_ | 0.001505 | 29.42_(1,57)_ | 0.000001 | 11.02_(1,74)_ | 0.001402 |
|  | Sex | 2.81_(1,57)_ | 0.099068 | 9.89_(1,74)_ | 0.002393 | 1.37_(1,57)_ | 0.246193 | 6.95_(1,74)_ | 0.010228 |
|  | G X S | 0.13_(1,57)_ | 0.717065 | 0.35_(1,74)_ | 0.554655 | 0.15_(1,57)_ | 0.698345 | 0.32_(1,74)_ | 0.576199 |
|  | *VC_line_* | 0.074(0.01) | <0.000001 | 0.11(0.02) | <0.000001 | 0.074(0.01) | <0.000001 | 0.11 (0.02) | <0.000001 |
|  | *VC_error_* | 0.007(0) | <0.000001 | 0.007(0) | <0.000001 | 0.007(0) | <0.000001 | 0.007 (0) | <0.000001 |
| RR | Gtyp | 12.16_(2,89)_ | 0.000021 | 5.15_(2,158)_ | 0.006826 | 11.93_(2,89)_ | 0.000026 | 5.06_(2,158)_ | 0.007442 |
|  | *VC_line_* | 0.041(0.01) | <0.000001 | 0.056(0.01) | <0.000001 | 0.041(0.01) | <0.000001 | 0.057 (0.01) | <0.000001 |
|  | *VC_error_* | 0.006(0) | <0.000001 | 0.006(0) | <0.000001 | 0.006(0) | <0.000001 | 0.006 (0) | <0.000001 |
| BC | Cross | 4.49_(1,107)_ | 0.036375 | 5.19_(1,137)_ | 0.02424 | 10.77_(1,106)_ | 0.001394 | 11.94_(1,137)_ | 0.000732 |
|  | Gtyp | 33.75_(1,107)_ | <0.000001 | 17.78_(1,137)_ | 0.000045 | 33.26_(1,106)_ | <0.000001 | 17.31_(1,137)_ | 0.000056 |
|  | C X G | 0.06_(1,107)_ | 0.807409 | 0.35_(1,137)_ | 0.554518 | 0.02_(1,106)_ | 0.900876 | 0.48_(1,137)_ | 0.491725 |
|  | Sex | 29.84_(1,106)_ | <0.000001 | 40.58_(1,134)_ | <0.000001 | 46.68_(1,106)_ | <0.000001 | 61.36_(1,134)_ | <0.000001 |
|  | C X S | 31.02_(1,106)_ | <0.000001 | 28.98_(1,134)_ | <0.000001 | 26.42_(1,106)_ | 0.000001 | 25.73_(1,134)_ | 0.000001 |
|  | G X S | 8.13_(1,106)_ | 0.005231 | 1.46_(1,134)_ | 0.22844 | 6.87_(1,106)_ | 0.010054 | 0.94_(1,134)_ | 0.334826 |
|  | C X G X S | 0.43_(1,106)_ | 0.515518 | 0.29_(1,134)_ | 0.593702 | 0.64_(1,106)_ | 0.426824 | 0.31_(1,134)_ | 0.580273 |
|  | *VC_line_* | 0.02(0) | <0.000001 | 0.024(0) | <0.000001 | 0.02(0) | <0.000001 | 0.023(0) | <0.000001 |
|  | *VC_error_* | 0.006(0) | <0.000001 | 0.007(0) | <0.000001 | 0.007(0) | <0.000001 | 0.008(0) | <0.000001 |
| KI | Cross | 37.31_(2,32)_ | <0.000001 |  |  | 36.91_(2,32)_ | <0.000001 |  |  |
|  | Gtyp | 15.31_(1,32)_ | 0.000447 |  |  | 15.72_(1,32)_ | 0.000387 |  |  |
|  | C X G | 0.05_(2,32)_ | 0.954467 |  |  | 0.04_(2,32)_ | 0.960262 |  |  |
|  | Sex | 20.24_(1,32)_ | 0.000085 |  |  | 25.33_(1,32)_ | 0.000018 |  |  |
|  | C X S | 32.03_(2,32)_ | <0.000001 |  |  | 33_(2,32)_ | <0.000001 |  |  |
|  | G X S | 0.02_(1,32)_ | 0.884448 |  |  | 0_(1,32)_ | 0.994781 |  |  |
|  | C X G X S | 0.44_(2,32)_ | 0.646036 |  |  | 0.34_(2,32)_ | 0.715022 |  |  |
|  | *VC_line_* | 0.018(0) | 0.000098 |  |  | 0.019(0.01) | 0.000087 |  |  |
|  | *VC_error_* | 0.003(0) | <0.000001 |  |  | 0.003(0) | <0.000001 |  |  |

a. As described in Materials and Methods, the principal components were calculated for the datasets individually (separately) or for all the data concatenated (jointly). The exception is the Inbred datasets, where the PC’s were extracted for the NC and CA populations together, but the data divided prior to analysis.

b. The genotype of site T30200C, where “partial” refers to the previously genotyped sample (Palsson and Gibson 2004), while “extended” indicates analysis conducted with the regenotyped data. Does not apply to the KI alleles, which were not regenotyped.

c. Depending on terms either F statistics or estimated variance components are tabulated. The F-statistic are reported with numerator and denominator degrees of freedom in brackets. Estimated variance components are listed with standard errors in brackets, both numbers multiplied by 1000 for visualization purposes. Significance of Variance components is derived from the *z*-distribution.
