# Supplementary material for: Tests for the replication of an association between Egfr and natural variation in Drosophila melanogaster wing morphology
Source: BMC Genet. 2005 Aug 15;6:44. doi: 10.1186/1471-2156-6-44 (PMC1208880; doi:10.1186/1471-2156-6-44)
Supplement: Additional Table 2 — Genotypic effects of T30200C on the first PC of the central region of the wing. This table illustrates the genotypic effects (and standard errors) for the T30200C association to wing shape of the central region. [file 1471-2156-6-44-S2.doc]

# Additional files

## Additional Table 2. Genotypic effects (and standard errors) of T30200C on the first PC of the central region of the wing.

|  |  | Partial sample ^b^ | | | Extended sample ^b^ | | |
| --- | --- | --- | --- | --- | --- | --- | --- |
|  | Exp ^a^ | CC | CT | TT | CC | CT | TT |
| Fem | INB CA | -0.0123(0.0038) |  | -0.0035(0.0022) | -0.0034(0.003) |  | -0.0039(0.0019) |
|  | INB NC | -0.0085(0.0022) |  | 0.0031(0.0014) | -0.0068(0.0022) |  | 0.0006(0.0011) |
|  | INB RR | -0.0123(0.0027) |  | 0.0034(0.0014) | -0.0087(0.003) |  | 0.0014(0.0015) |
|  | INB BC | -0.0108(0.0023) |  | 0.0036(0.0014) | -0.0081(0.0026) |  | 0.0013(0.0014) |
|  | RR | -0.0055(0.0033) | -0.0011(0.0011) | 0.0051(0.0009) | -0.0002(0.0031) | -0.0008(0.001) | 0.0032(0.0008) |
|  | BC C144 |  | -0.0014(0.0013) | 0.0052(0.0009) |  | -0.0008(0.0014) | 0.0045(0.0008) |
|  | BC C25 |  | 0.0024(0.0013) | 0.01(0.0008) |  | 0.0041(0.0014) | 0.0085(0.0007) |
|  | KI Sam |  |  |  | -0.0015(0.0017) |  | 0.0045(0.0019) |
|  | KI bs |  |  |  | 0.0067(0.0017) |  | 0.012(0.0019) |
|  | KI E1 |  |  |  | -0.006(0.0017) |  | -0.0008(0.0019) |
| Male | INB CA | -0.0149(0.0038) |  | -0.0054(0.0022) | -0.0067(0.003) |  | -0.0064(0.0019) |
|  | INB NC | -0.01(0.0022) |  | 0.0016(0.0014) | -0.0091(0.0022) |  | -0.001(0.0011) |
|  | INB RR | -0.0126(0.0027) |  | 0.0024(0.0014) | -0.0107(0.003) |  | 0.0002(0.0015) |
|  | INB BC | -0.0115(0.0023) |  | 0.0025(0.0014) | -0.0101(0.0026) |  | -0.0001(0.0014) |
|  | BC C144 |  | -0.0006(0.0013) | 0.0044(0.0009) |  | -0.0009(0.0014) | 0.0038(0.0008) |
|  | BC C25 |  | -0.0005(0.0013) | 0.0045(0.0008) |  | 0.0003(0.0014) | 0.0033(0.0007) |
|  | KI Sam |  |  |  | -0.0075(0.0017) |  | -0.0015(0.0019) |
|  | KI bs |  |  |  | 0.0087(0.0017) |  | 0.0133(0.0019) |
|  | KI E1 |  |  |  | -0.0076(0.0017) |  | -0.0013(0.0019) |

a. The effects were estimated across populations and subsets of data. A subset of NC lines that were used for crosses (INB RR and INB BC), the two BC test crosses to NC144 and NC025 (BC C144 and BC C25), and the KI test crosses to Sam, *bs* and *Egfr^Ellipse^* (*E1*) chromosomes.

b. The genotype of T30200C for previously genotyped lines (partial) and all lines after regenotyping.
